# Association Between Weight‐Adjusted Waist Index and Depressive Symptoms Among Middle‐Aged and Older Adults: Evidence From Two Prospective Longitudinal Cohort Studies

**DOI:** 10.1111/cns.70496

**Published:** 2025-07-01

**Authors:** Xingjun Chen, Jianming Xu, Li Wen, Haiyan Deng, Xiaoxi Lu, Guangyan Liu

**Affiliations:** ^1^ Affiliated Hospital of Guangdong Medical University Zhanjiang Guangdong China

**Keywords:** aging, depression, ELSA, HRS, prospective cohort study, weight‐adjusted waist index

## Abstract

**Aim:**

The association between obesity and depression has been debated. This study aimed to explore the long‐term relationship between the weight‐adjusted waist index (WWI) and depression in different ethnic groups.

**Methods:**

This prospective cohort study analyzed data from English Longitudinal Study on Aging (ELSA) and Health and Retirement Study (HRS). The exposure variable was the WWI at baseline, calculated by dividing the waist circumference (cm) by the square root of weight (kg). Depressive symptoms were assessed using the CESD‐8. The longitudinal relationship between WWI and depression was analyzed using Cox proportional hazards and restricted cubic spline (RCS) regression models.

**Results:**

During the 12‐year follow‐up period, depressive symptoms were observed in 55.1% of HRS patients (1851/3359) and 54.8% of ELSA patients (1810/3303). In fully adjusted Cox regression analysis, participants in the 4th quartile of the WWI exhibited a 33% elevated risk of depression (HR = 1.33, 95% CI: 1.15–1.54). Furthermore, the fully adjusted RCS regression model revealed a positive linear association between WWI and the risk of depression.

**Conclusion:**

Our studies demonstrated a positive linear correlation between WWI and elevated risk of depression. Alterations in WWI have the potential to predict the occurrence of depression in middle‐aged and elderly individuals.

## Graphical abstract

Our results point to the importance of the WWI in predicting the risk of depression in middle‐aged and older adults. In future intervention wording, WWI may become a target to reduce the high prevalence of depression in middle‐aged and older adults. Image was created in BioRender. Liu, G. (2024) https://BioRender.com/e47a501.

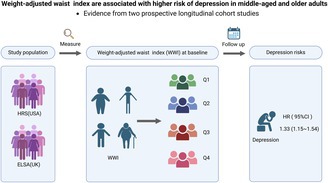

## 1 Introduction

Depression is a significant global mental health disorder, characterized by a range of symptoms, including low mood, lack of interest, cognitive decline, and potential suicidal tendencies [1, 2]. Furthermore, it may also increase the risk of developing cardiovascular and cerebrovascular diseases [3]. This situation not only diminishes the quality of personal life, but also imposes a significant emotional and economic burden on families and society [4]. Current estimates suggest that approximately 300 million individuals worldwide are affected by depression; moreover, both the prevalence and impact of the disorder have been rising each year [5]. The prevalence of depression is particularly elevated in the elderly population, in whom it frequently occurs concomitantly with other chronic diseases, resulting in increased disability and mortality rates [6]. The pathogenesis of depression is characterized by considerable complexity, involving a diverse array of factors such as genetics, environment, and biology [7, 8, 9]. This intricate interplay contributes to substantial heterogeneity and uncertainty in clinical presentations, therapeutic strategies, and prognostic outcomes [10]. Hence, enhancing the early detection and prevention of depression among the general population has emerged as a pressing concern for global public health and medical attention.

Obesity is a pathological condition characterized by the abnormal buildup of body fat, which functions as a precursor to numerous metabolic‐related illnesses [11]. In addition to being implicated in the occurrence and progression of various chronic diseases, obesity is also strongly associated with an increased risk of mental disorders [12, 13, 14]. While controversy persists regarding the impact of obesity on depression, most studies indicate that obese individuals are at an increased risk of depression [15, 16]. However, some studies have reported findings consistent with the “Jolly Fat” hypothesis, suggesting that obese individuals may exhibit lower levels of depressive symptoms [17, 18] Furthermore, other studies have indicated a nonlinear U‐shaped correlation between weight and the probability of experiencing depressive symptoms [19, 20]. This inconsistency may be attributable to the widespread use of body mass index (BMI) as a measure of obesity in these studies. Furthermore, the “obesity paradox,” highlights a counterintuitive finding in which elderly individuals with certain diseases who are overweight or obese may experience better outcomes than those with normal weight or underweight [21], thus challenging the validity of conventional obesity measures, such as BMI and waist circumference (WC). BMI is incapable of differentiating between fat and muscle mass and fails to accurately reflect the distribution of adipose tissue within the body [22, 23]. Likewise, waist circumference, a conventional indicator of obesity, also has its own constraints [24]. Park et al. recently introduced a new obesity index known as the weight‐adjusted waist index (WWI), which is calculated by dividing an individual's waist by the square root of their weight [25]. Compared to BMI, WWI more accurately reflects body composition characteristics, including high fat mass, muscle loss, and osteoporosis [26]. Recent studies have indicated that WWI may exhibit greater sensitivity than traditional BMI and WC in predicting depressive symptoms. Indeed, one cross‐sectional study found a positive association between WWI and depressive symptoms, which was stronger than the association between BMI and WC [27, 28] However, due to its cross‐sectional design, this study did not account for dynamic changes in depressive symptoms. Additionally, a recent Chinese cohort study suggested that WWI is a better predictor of depression in middle‐aged and older adults than BMI [29]. Nevertheless, this study was limited to the Chinese population, which restricts the generalizability of the findings. The association between WWI and depression in middle‐aged and older populations in different countries currently remains unknown.

In this context, the present study used nationally representative samples from the English Longitudinal Study of Aging (ELSA) and Health and Retirement Study (HRS) to examine the relationship between WWI and depression among middle‐aged and older adults in the United Kingdom and United States. These findings thus provide preliminary evidence supporting the cross‐population applicability of WWI as an observational indicator of the risk of depression.

## 2 Methods

### 2.1 Study Population

**FIGURE 1:**
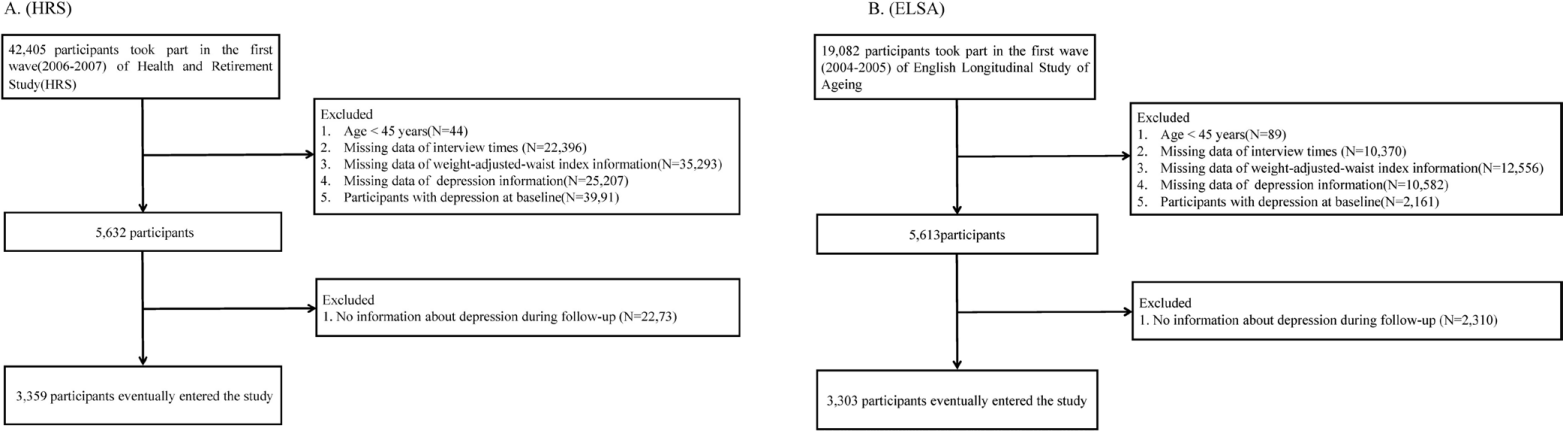
Flowchart of the screening of the study populations for HRS (A) and ELSA (B).

The data used in this study were sourced from two nationally representative prospective cohorts, specifically community‐dwelling adults aged over 45 years, in both the United States and the United Kingdom [30, 31]. HRS was approved by the University of Michigan Institutional Review Board and the National Institute on Aging (HUM 00061128). ELSA was approved by the Multicenter Research Ethics Committee of London (MREC/01/2/91). All participants in both cohorts signed informed consent forms to participate. We established Wave 2 of the ELSA (2004–2005) and Wave 8 of the HRS (2006–2007) as the baselines (Figure 1). The follow‐up period extended from Waves 3 to 9 for the ELSA, and from Waves 9 to 15 for the HRS. After excluding individuals aged < 45 years, those with missing WWI information during the follow‐up period, those with missing depressive symptoms, and those with depression or missing visits at baseline, 3359 and 3303 participants were enrolled in the 12‐year follow‐up of the HRS and ELSA, respectively.

### 2.2 Weight‐Adjusted Waist Index

WWI was calculated by dividing the WC (in cm) by the square root of the weight (in kg) [25]. Trained medical professionals performed measurements using professional‐grade equipment to ensure accuracy. During Wave 8 of the HRS (2006), participants were instructed to stand upright, point to their navel, and have their waist measured by wrapping a tape measure around their waist at the level of the navel [32]. In each survey, participants' waist circumferences were measured once, and recorded to the nearest quarter inch. In Wave 2 of the ELSA (2004), the midpoint between the participant's lower ribs and the upper edge of the iliac crest was measured using a tape measure, and the average of the two valid measurements was recorded as the WC measurement value. In both the HRS and ELSA studies, respondents' net weight was measured using a portable electronic scale.

### 2.3 Depressive Symptoms

In the two cohort studies, the eight‐item Center for Epidemiological Research Depression Scale (CESD‐8) was applied to assess depressive symptoms in older adults. Although the CESD‐8 does not meet the diagnostic precision required for clinical diagnosis, it has repeatedly been employed as a depression‐screening tool in numerous published studies [33, 34, 35]. Moreover, in the original study, the items on the CESD‐8 were administered via standardized interviews, and recorded by trained researchers. The CESD‐8 encompasses cognitive‐emotional symptoms, including “feeling depressed,” “feeling lonely” and “feeling sad” alongside somatic symptoms such as “everything is an effort,” “troubled sleep” and “not being able to sleep.” Additionally, two positive symptoms were assessed: “feeling happy” and “enjoying life.” During the biennial follow‐up survey, participants were asked about the occurrence of eight depressive symptoms in the preceding week, with responses recorded as yes or no. The cumulative scores ranged from 0 to 8 (yes = 1; no = 0). Drawing on previous validation studies, a CESD‐8 score ≥ 3 has been shown to reliably identify clinically significant depressive symptoms in older adults. Therefore, this threshold was applied to detect notable depressive symptoms [36].

### 2.4 Covariates

The covariates in this study included sociodemographic characteristics, such as age, sex, education level, marital status, chronic diseases (yes/no hypertension or diabetes), and lifestyle factors such as smoking status and alcohol consumption. Measurements, including WC, height, weight, systolic blood pressure (SBP) and diastolic blood pressure (DBP) were recorded using calibrated instruments according to standardized procedures. Based on the consistency of the covariate classification between the HRS and ELSA, education level was categorized into two groups (junior high school and below or high school or above). Marital status was categorized as either married or partnered and other marital statuses (unmarried, separated, divorced, or widowed). Smoking status was categorized as smoker (both previous and current smokers) or never smoked. Drinking status was evaluated based on the frequency of drinking in the past 12 months and was similarly grouped into drinkers (including previous and current drinkers) and never‐drinkers.

### 2.5 Handling of Missing Variables

Table S1 presents detailed information on the missing data for HRS and ELSA in this study. To ensure the maximum sample size, we implemented multiple imputations for missing covariate data, aiming to closely simulate the actual data [37].

### 2.6 Statistical Analysis

In this study, participants were categorized into four groups (Q1–Q4) based on WWI quartiles. All continuous variables were assessed for normality using the Kolmogorov–Smirnov test. Asymptotic significance (*p* < 0.001) indicated a statistically significant deviation from the normal distribution. For continuous variables that followed a normal distribution, the mean ± standard deviation (x ± s) was employed for statistical description, while the analysis of variance (ANOVA) was applied to evaluate intergroup differences. For continuous variables that did not follow a normal distribution, data are presented as the medians with interquartile ranges (IQR), while the Kruskal–Wallis H test was employed to assess differences between the groups. Categorical variables were characterized by frequencies and percentages, while differences between groups were assessed using the chi‐square (Χ^2^) test. Univariate and multivariate Cox regression models were employed for prospective analysis of the correlation between WWI and depression throughout the follow‐up period, while the chi‐square tests were applied to assess this trend. We further performed stratification and interaction analyses based on sex, age, drinking habits, smoking status, educational level, marital status, and presence of chronic diseases. Furthermore, restricted cubic spline (RCS) regression of the hazard ratio (HR) was employed to delve deeper into the potential nonlinear correlation between WWI and depression. To ensure the robustness of the results, a sensitivity analysis was conducted, excluding multiple imputations (Tables S2 and S3). All statistical analyses were conducted using *R* software (version 4.4.2), and two‐sided *p* value < 0.05 was considered statistically significant.

## 3 Results

### 3.1 Participant Baseline Characteristics by WWI Quartiles

The final cohort comprised 3359 and 3303 respondents for HRS and ELSA, most of whom were female, with a mean age of 67.8 ± 8.1 years (HRS) and 64.3 ± 8.4 years (ELSA). The baseline characteristics are presented in Table 1. Compared with respondents in the WWI Q1 group, those with a higher WWI value had an older age, more significant smoking history, elevated systolic blood pressure, increased BMI, and greater susceptibility to complications such as hypertension and/or diabetes. Conversely, respondents in the WWI Q4 group were more likely to have a higher level of education and history of alcohol consumption than those in the WWI Q1 group. Over the course of the 12‐year follow‐up period, 1851 participants from the HRS cohort developed depression, with an incidence rate of 55.1% (1851/3359 participants). Similarly, 54.8% of the respondents in the ELSA cohort exhibited depressive symptoms (1810/3303).

**TABLE 1:** Baseline characteristics of the study population in HRS and ELSA.

Variables	HRS				
	Q1 (*n* = 840)	Q2 (*n* = 839)	Q3 (*n* = 840)	Q4 (*n* = 840)	*p*
Age, (years)	65.0 (60.0, 70.0)	67.0 (62.0, 72.0)	68.0 (63.0, 73.0)	70.0 (65.0, 76.0)	< 0.001
Sex, *n* (%)					
Male	576 (68.6)	413 (49.2)	416 (49.5)	592 (70.5)	< 0.001
Female	264 (31.4)	426 (50.8)	424 (50.5)	248 (29.5)	
Education level, *n* (%)					
Junior high school and below	45 (5.4)	100 (11.9)	133 (15.8)	184 (21.9)	< 0.001
High school or above	795 (94.6)	739 (88.1)	707 (84.2)	656 (78.1)	
Marital status, *n* (%)					
Other marital status	170 (20.2)	171 (20.4)	186 (22.1)	303 (36.1)	< 0.001
Married or partnered	670 (79.8)	668 (79.6)	654 (77.9)	537 (63.9)	
Smoking status, *n* (%)					
No	416 (49.8)	392 (46.9)	340 (40.6)	362 (43.5)	< 0.001
Yes	419 (50.2)	443 (53.1)	497 (59.4)	471 (56.5)	
Drinking status, *n* (%)					
No	299 (35.6)	333 (39.7)	369 (43.9)	429 (51.1)	< 0.001
Yes	541 (64.4)	506 (60.3)	471 (56.1)	411 (48.9)	
SBP, (mmHg)	124.5 (113.6, 138.0)	127.7 (116.3, 140.7)	128.7 (117.6, 142.7)	129.8 (117.0, 144.3)	< 0.001
DBP, (mmHg)	79.0 (71.9, 86.7)	79.3 (72.3, 87.2)	79.3 (73.0, 87.0)	79.0 (71.3, 86.3)	0.681
Height, (m)	1.7 (1.6, 1.7)	1.7 (1.6, 1.8)	1.7 (1.6, 1.7)	1.6 (1.5, 1.7)	< 0.001
Weight, (Kg)	73.6 (63.5, 85.3)	80.7 (69.9, 92.9)	82.8 (72.1, 95.5)	81.0 (70.1, 94.6)	< 0.001
BMI, (kg/m^2^)	26.2 (23.6, 29.2)	28.1 (25.1, 31.3)	29.4 (26.5, 33.2)	30.8 (27.6, 35.4)	< 0.001
Waist, (cm)	86.4 (78.7, 94.0)	96.5 (90.2, 104.1)	102.9 (96.5, 111.1)	109.9 (101.6, 119.4)	< 0.001
Hypertension, *n* (%)					
No	550 (65.5)	459 (54.7)	410 (48.8)	298 (35.5)	< 0.001
Yes	290 (34.5)	380 (45.3)	430 (51.2)	542 (64.5)	
Diabetes, *n* (%)					
No	795 (94.6)	730 (87)	698 (83.1)	638 (76)	< 0.001
Yes	45 (5.4)	109 (13)	142 (16.9)	202 (24)	
WWI	10.1 (9.8, 10.3)	10.8 (10.7, 10.9)	11.3 (11.2, 11.4)	12.1 (11.8, 12.5)	< 0.001

Variables	ELSA				
	Q1 (*n* = 826)	Q2 (*n* = 825)	Q3 (*n* = 826)	Q4 (*n* = 826)	*p*
Age, (years)	61.0 (56.0, 67.0)	61.0 (57.0, 69.0)	63.0 (57.0, 70.0)	67.0 (60.0, 74.0)	< 0.001
Sex, *n* (%)					
Male	628 (76)	477 (57.8)	376 (45.5)	366 (44.3)	< 0.001
Female	198 (24)	348 (42.2)	450 (54.5)	460 (55.7)	
Education level, *n* (%)					
Junior high school and below	191 (26)	246 (32.7)	299 (38.8)	387 (51.6)	< 0.001
High school or above	543 (74)	507 (67.3)	471 (61.2)	363 (48.4)	
Marital status, *n* (%)					
Other marital status	207 (26)	207 (25.9)	196 (24.5)	235 (28.9)	0.219
Married or partnered	590 (74)	593 (74.1)	605 (75.5)	577 (71.1)	
Smoking status, *n* (%)					
No	396 (47.9)	347 (42.1)	299 (36.2)	271 (32.8)	< 0.001
Yes	430 (52.1)	478 (57.9)	527 (63.8)	554 (67.2)	
Drinking status, *n* (%)					
No	55 (7)	52 (6.7)	49 (6.4)	76 (10.2)	0.019
Yes	729 (93)	722 (93.3)	718 (93.6)	670 (89.8)	
SBP, (mmHg)	74.0 (68.0, 81.0)	75.7 (68.7, 83.0)	77.3 (70.1, 84.7)	76.7 (69.0, 84.0)	< 0.001
DBP, (mmHg)	110.0 (102.1, 119.7)	112.7 (103.3, 122.7)	115.7 (106.7, 126.3)	116.0 (107.3, 126.7)	< 0.001
Height, (m)	1.6 (1.6, 1.7)	1.7 (1.6, 1.7)	1.7 (1.6, 1.7)	1.6 (1.6, 1.7)	< 0.001
Weight, (Kg)	68.0 (60.7, 77.4)	75.2 (65.7, 84.5)	79.0 (70.4, 88.9)	81.0 (71.1, 92.5)	< 0.001
BMI, (kg/m^2^)	24.9 (22.7, 27.1)	26.7 (24.6, 29.5)	28.1 (25.8, 31.2)	29.7 (26.7, 32.9)	< 0.001
Waist, (cm)	82.3 (76.6, 88.3)	92.2 (86.2, 98.2)	98.9 (93.0, 105.2)	105.5 (98.8, 113.4)	< 0.001
Hypertension, *n* (%)					
No	631 (76.4)	548 (66.4)	498 (60.3)	434 (52.5)	< 0.001
Yes	195 (23.6)	277 (33.6)	328 (39.7)	392 (47.5)	
Diabetes, *n* (%)					
No	804 (97.3)	791 (95.9)	774 (93.7)	730 (88.4)	< 0.001
Yes	22 (2.7)	34 (4.1)	52 (6.3)	96 (11.6)	
WWI	9.9 ± 0.4	10.7 ± 0.1	11.1 ± 0.1	11.8 ± 0.4	< 0.001

Abbreviations: BMI, body mass index; DBP, diastolic blood pressure; SBP, systolic blood pressure; WWI, weight‐adjusted waist index.

### 3.2 Univariate Analysis of the Risk of Depression

As presented in Table 2, the univariate analysis conducted on the HRS and ELSA participants revealed significant associations between depression and various factors such as age, sex, education level, marital status, smoking status, alcohol consumption, SBP, BMI, hypertension, and diabetes. Notably, WWI demonstrated a significant positive correlation with depression in both HRS (HR = 1.35, 95% CI: 1.29–1.42, *p* < 0.001) and ELSA (HR = 1.33, 95% CI: 1.24–1.42, *p* < 0.001).

**TABLE 2:** Results of the univariate analysis of depression.

Variables	HRS		ELSA	
	HR (95% CI)	*p*	HR (95% CI)	*p*
Age, (years)	1.06 (1.05–1.06)	< 0.001	1.05 (1.05–1.06)	< 0.001
Male, *n* (%)	1.31 (1.19–1.44)	< 0.001	1.50 (1.36–1.65)	< 0.001
High school or above, *n* (%)	0.52 (0.47–0.59)	< 0.001	0.56 (0.51–0.61)	< 0.001
Married or partnered, *n* (%)	0.71 (0.64–0.78)	< 0.001	0.64 (0.58–0.71)	< 0.001
Smoking status, *n* (%)				
No	Reference	< 0.001	Reference	< 0.001
Yes	1.19 (1.09–1.30)		1.19 (1.08–1.30)	
Drinking status, *n* (%)				
No	Reference	< 0.001	Reference	< 0.001
Yes	0.79 (0.72–0.87)		0.66 (0.56–0.77)	
SBP, (mmHg)	1.01 (1.00–1.01)	< 0.001	1.010 (1.004–1.009)	< 0.001
DBP, (mmHg)	0.999 (0.995–1.003)	0.539	0.992 (0.988–0.997)	< 0.001
Height, (m)	0.11 (0.07–0.18)	< 0.001	0.06 (0.04–0.11)	< 0.001
Weight, (Kg)	0.996 (0.993–0.998)	0.002	0.995 (0.992–0.998)	0.001
BMI, (kg/m^2^)	1.010 (1.002–1.018)	0.022	1.02 (1.01–1.03)	< 0.001
Waist, (cm)	1.006 (1.003–1.009)	< 0.001	1.0030 (0.9995–1.0067)	0.09
Hypertension, *n* (%)				
No	Reference	< 0.001	Reference	< 0.001
Yes	1.36 (1.24–1.49)		1.39 (1.26–1.52)	
Diabetes, *n* (%)				
No	Reference	< 0.001	Reference	< 0.001
Yes	1.46 (1.30–1.65)		1.82 (1.54–2.15)	
WWI	1.35 (1.29–1.42)	< 0.001	1.33 (1.24–1.42)	< 0.001

Abbreviations: BMI, body mass index; DBP, diastolic blood pressure; SBP, systolic blood pressure; WWI, weight‐adjusted waist index.

### 3.3 Multivariate Analysis of WWI and the Risk of Depression

To assess the correlation between WWI and depression, we constructed three Cox proportional hazards models, as outlined in Table 3. Model 2 incorporated adjustments for age and sex. Model 3, built on Model 2, included additional covariates for adjustment for education level, alcohol consumption, smoking habits, marital status, height, SBP, DBP, and diabetes. In HRS, for every unit increase in WWI, the risk of depression rose by 35% (HR = 1.35, 95% CI: 1.29–1.42), 21% (HR = 1.21, 95% CI: 1.15–1.28), and 11% (HR = 1.11, 95% CI: 1.05–1.18) in model 1, 2, and 3, respectively. Likewise, in ELSA, the depression risk increased by 33% (Model 1, HR = 1.33, 95% CI: 1.24–1.42), 25% (Model 1, HR = 1.25, 95% CI: 1.17–1.34), and 18% (Model 1, HR = 1.18, 95% CI: 1.10–1.27) per unit increase in WWI.

**TABLE 3:** Multivariable‐adjusted HR and 95% CI of the WWI quartiles associated with depression.

(HRS) variables	Model 1		Model 2		Model 3	
	HR (95% CI)	*p*	HR (95% CI)	*p*	HR (95% CI)	*p*
WWI	1.35 (1.29–1.42)	< 0.001	1.21 (1.15–1.28)	< 0.001	1.11 (1.05–1.18)	< 0.001
1st Quartile (< 10.520)	1 (Reference)		1 (Reference)		1 (Reference)	
2nd Quartile (10.520–11.041)	1.19 (1.03–1.37)	0.015	1.15 (0.99–1.32)	0.06	1.07 (0.93–1.23)	0.357
3rd Quartile (11.041–11.606)	1.51 (1.32–1.72)	< 0.001	1.40 (1.22–1.6)	< 0.001	1.22 (1.06–1.4)	0.006
4th Quartile (≥ 11.606)	2.06 (1.81–2.35)	< 0.001	1.60 (1.40–1.84)	< 0.001	1.33 (1.15–1.54)	< 0.001
*p* for trend	1.28 (1.23–1.33)	< 0.001	1.18 (1.13–1.23)	< 0.001	1.10 (1.06–1.16)	< 0.001

(ELSA) variables	Model 1		Model 2		Model 3	
	HR (95% CI)	*p*	HR (95% CI)	*p*	HR (95% CI)	*p*
WWI	1.33 (1.24–1.42)	< 0.001	1.25 (1.17–1.34)	< 0.001	1.18 (1.1–1.27)	< 0.001
1st Quartile (< 10.394)	1 (Reference)		1 (Reference)		1 (Reference)	
2nd Quartile (10.394–10.89)	1.05 (0.92–1.2)	0.489	1.08 (0.94–1.24)	0.295	1.05 (0.91–1.2)	0.526
3rd Quartile (10.89–11.374)	1.18 (1.03–1.35)	0.017	1.22 (1.07–1.4)	0.004	1.16 (1.01–1.34)	0.039
4th Quartile (≥ 11.374)	1.62 (1.43–1.85)	< 0.001	1.50 (1.31–1.71)	< 0.001	1.33 (1.15–1.54)	< 0.001
*p* for trend	1.18 (1.13–1.23)	< 0.001	1.15 (1.1–1.2)	< 0.001	1.10 (1.05–1.16)	< 0.001

*Note:* Model 1: unadjusted. Model 2: adjusted for age and sex. Model 3: adjusted for Model 1 + education level, marital status, smoking status, drinking status, SBP, DBP, height, diabetes.

Abbreviations: DBP, diastolic blood pressure; SBP, systolic blood pressure; WWI, weight‐adjusted waist index.

Subsequently, we segmented WWI into quartiles, using these as thresholds for the regression analysis of depression. In the comprehensive Model 3, when benchmarked against the 1st quartile, the HR values progressively increased for the 2nd, 3rd, and 4th quartiles. Specifically, in HRS and ELSA, individuals falling within the 4th quartile of WWI exhibited a notable 33% elevation in depression risk (HR = 1.33, 95% CI:1.15–1.54). Across all models, the *p* value for the trend test was consistently below 0.001, confirming a strong linear correlation between WWI and depression.

### 3.4 Stratified Analysis

**FIGURE 2:**
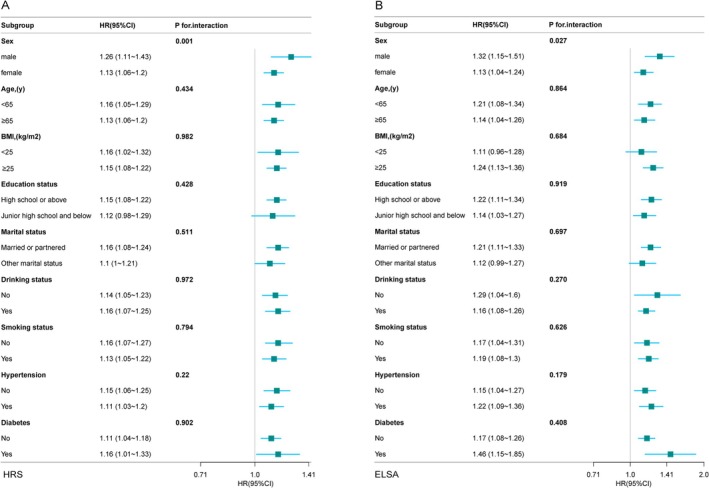
Subgroup analysis of the association between weight‐adjusted waist index and depression risk in the HRS (A) and ELSA (B) cohorts. Subgroups were defined based on sex, age, BMI, education level, smoking status, alcohol consumption, marital status, hypertension, and diabetes.

Groups were stratified by age, sex, BMI, smoking status, drinking status, marital status, hypertension, diabetes mellitus, and depression and analyzed based on their interactions. Within the HRS and ELSA datasets, an interaction between WWI and depression was observed, primarily varying across genders (Figure 2; HRS: P for interaction = 0.001; ELSA: P for interaction = 0.027). Furthermore, a significant positive correlation between WWI and depression persisted within the subgroups stratified by age, drinking status, smoking status, hypertension, and diabetes.

### 3.5 Curve Fitting

**FIGURE 3:**
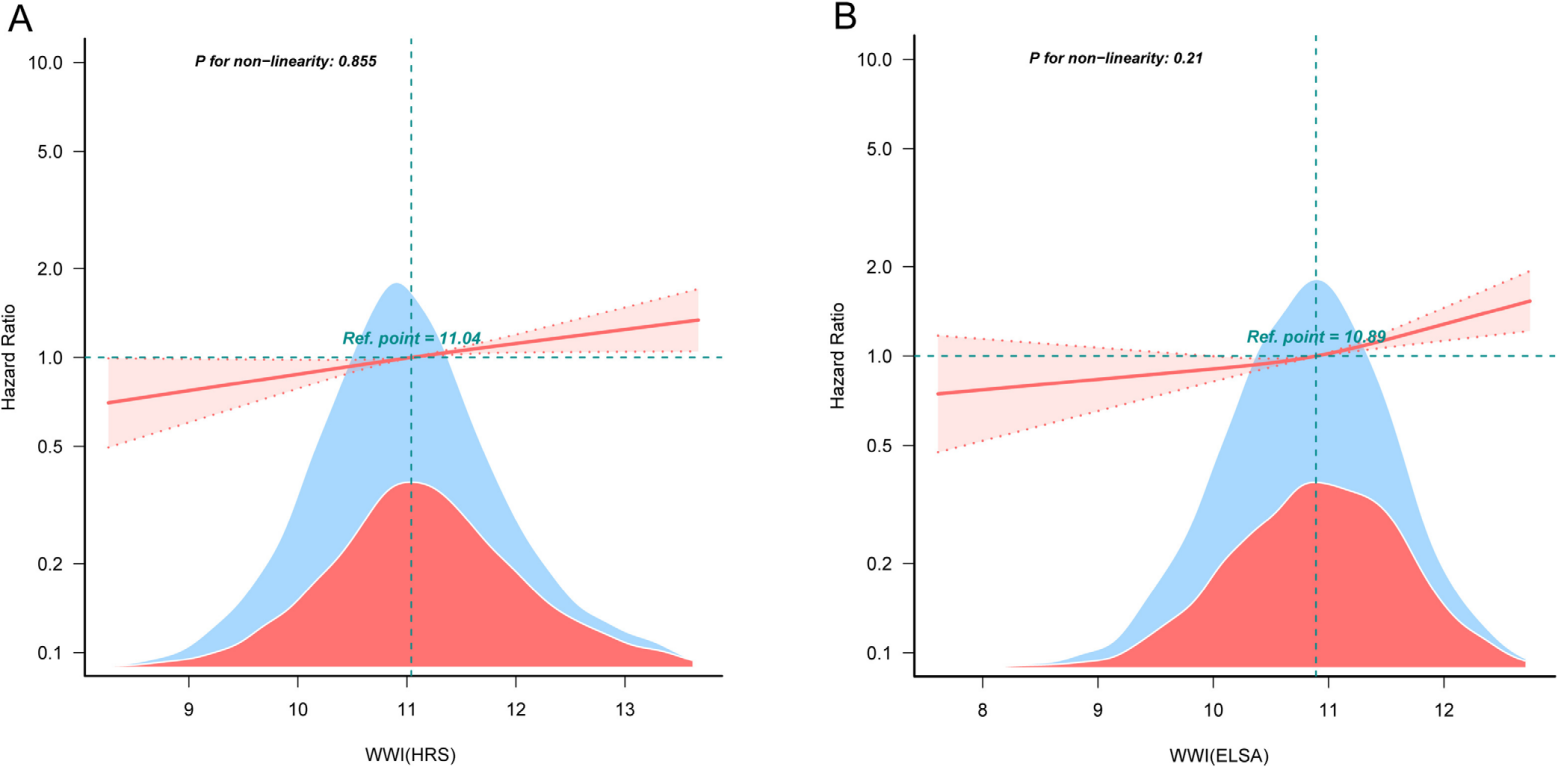
RCS regression analysis of the association between WWI and depression for HRS (A) and ELSA (B). The regression model adjusted for age, gender, smoking status, alcohol consumption, education level, marital status, height, systolic blood pressure, diastolic blood pressure, and diabetes.

We further examined the correlation between WWI and depression risk using the RCS Cox proportional hazard regression model. The results (Figure 3A,B) revealed a noticeable positive linear association between WWI and depression risk in the fully adjusted RCS regression model (HRS: P‐non‐linear = 0.86; ELSA: P‐non‐linear = 0.21).

## 4 Discussion

In this prospective study, for the first time, we examined patient data collected over a follow‐up period of up to 12 years from the HRS and ELSA cohorts to investigate the longitudinal relationship between WWI and depression in different ethnic groups. After rigorously adjusting for multiple covariates, we focused on individuals who were not depressed at baseline in this longitudinal association analysis. Our findings revealed a significant positive correlation between WWI and depressive symptoms in both the HRS and ELSA cohorts. This correlation persisted even when WWI was categorized into quartiles (Q1–Q4) for further analyses. Additional stratified analyses confirmed the resilience of this positive correlation across various populations. These results suggest that WWI could serve as a valuable indicator for the early identification of depressive symptoms, and that evaluating WWI may aid in predicting future depression risk among older adults. This study provides further evidence that obesity plays a role in the onset and progression of senile depression across ethnic groups.

Obesity and depression are both major global health concerns. Despite numerous investigations into this relationship, a definitive consensus regarding the link between these conditions remains elusive [14, 38]. Globally, the primary established criteria for defining obesity are BMI and WC. A prospective cohort study, encompassing 6514 middle‐aged and elderly Americans, revealed a direct correlation between obesity (measured via BMI) and the emergence of depressive symptoms [39]. Comparable patterns emerge from multiple cohort analyses within the UK biospecimen database [40]. Additionally, novel obesity indicators, such as the sagittal abdominal diameter, have demonstrated a positive correlation with the risk of depression [41]. Curiously, WC, a marker of central obesity, bears no significant association with depression [42]. Conversely, one cohort study among 3377 elderly Chinese individuals yields a contrasting finding: men with obesity (BMI ≥ 28.0 kg/m^2^) or abdominal obesity (WC ≥ 85 cm) appear less susceptible to depressive symptoms [43].

Following the definition of the obesity paradox, the reliability of BMI and WC as obesity markers has been investigated. This paradox challenges the conventional belief that obesity inexorably leads to a shorter lifespan [21, 44]. Discrepancies in research findings on obesity and depression may arise from the inability of BMI to differentiate between unfavorable bodily changes and metabolic states, such as reduced muscle mass and increased fat mass under specific conditions. While WC offers several advantages in assessing visceral fat accumulation, it falls short in accurately reflecting overall fat content [24]. In this context, WWI, a novel obesity metric, offers significant advantages in assessing the occurrence and mortality of heart failure [25]. However, standardizing WC/weight measurements circumvents the obesity paradox to some extent [45]. Furthermore, WWI is favored across various research domains due to its simplicity in calculating and distinguishing between muscle and fat mass. Currently, cross‐sectional studies on WWI and depression have suggested a positive correlation between the two, however, the limitations of the study design preclude inferring a causal relationship [27, 46, 47]. Furthermore, research has emphasized the longitudinal correlation between WWI and depressive symptoms among middle‐aged and older Chinese individuals [29]. In recent years, the relationship between obesity and depression has been found to vary based on several factors such as race and sex [48, 49]. This study, designed from this perspective, further establishes that WWI linearly elevates the risk of depression across ethnic groups. Consistent results from the smooth curve fitting indicated that as WWI increased, the risk of depression also increased.

Biological, psychological, and behavioral factors may influence the relationship between obesity and depressive symptoms. From a social psychology perspective, obese individuals may endure social discrimination and stigma due to their body size and associated health issues, which can increase their risk of developing depressive symptoms [50]. Biologically, immune cells infiltrate adipocytes in obese individuals, triggering an overproduction of pro‐inflammatory cytokines [51]. This, in turn can trigger neuroinflammation, disrupt neurotransmitter release, and hyperactivate the hypothalamus‐pituitary‐adrenal (HPA) axis, thereby increasing the risk of depressive symptoms [51]. Furthermore, HPA axis activation results in abnormally high cortisol levels and neuronal damage in the brain regions crucial for emotion regulation, such as the hippocampus and amygdala, potentially exacerbating depression [52, 53]. Additionally, alterations in the gut microbiota and metabolite composition in obese individuals may influence the brain functions linked to depressive behaviors via the gut‐brain axis [54, 55].

Stratified analyses have indicated that the association between WWI and the risk of depression was stronger among middle‐aged and older male participants compared with women. This agrees with the results of several prior studies which have found that visceral fat accumulation is more common in middle‐aged and older men than in women [26]. This disparity may be explained by dysregulation of sex hormone modulation. Testosterone appears to exert a protective effect against depression in men; testosterone deficiency can reduce the firing rate of serotonergic neurons, thereby decreasing the efficacy of monoaminergic neurotransmission in the brain [56]. Moreover, a low testosterone state decreases dopamine activity in neural pathways [57, 58], and is often accompanied by reduced serum vitamin D concentrations, which increases the likelihood of neuronal oxidative damage [59, 60]. Furthermore, social factors should not be overlooked, as men often face various life stressors and are more prone to engage in health risk‐inducing behaviors such as smoking, alcohol abuse, and physical inactivity, as well as experiencing stigma associated with mental health issues, all of which may further amplify the relationship between WWI and depression.

The strength of this study lies in its prospective design, which utilized follow‐up data from two large cohorts of middle‐aged and elderly individuals. This enabled us to explore, for the first time, the association between WWI and depressive symptoms across different ethnic populations. Furthermore, we evaluated this association using both continuous and categorical variables, while controlling for multiple potential confounders. Additionally, we conducted subgroup and sensitivity analyses to ensure the robustness of our findings, and further examined the association between WWI and risk of depressive symptoms, ultimately determining a positive linear relationship. However, this study has several limitations. First, despite efforts to minimize bias, we cannot fully eliminate the influence of other potential confounding factors, including genetic components, lifestyle factors (e.g., diet and physical activity), psychosocial factors (e.g., life events and stress), and other medical conditions. Second, the diagnosis of depressive symptoms relied on self‐reports from middle‐aged and elderly participants, which may introduce inherent recall bias. Third, there is currently no standardized threshold for defining obesity according to the WWI, thereby necessitating further analyses with larger and more representative samples. Furthermore, the data analyzed in this study did not encompass individuals from all age groups or racial backgrounds, underscoring the need for future studies to further broaden the diversity of participants in terms of both age and race. Finally, although we excluded individuals with depression at baseline, the possibility of reverse causation, whereby depressive symptoms affect WWI, cannot be discounted. Complex models and long‐term data are required for verification.

## 5 Conclusions

The present analysis of two cohort studies demonstrated a positive correlation between WWI and increased occurrence of depression among middle‐aged and elderly individuals. The variation in WWI serves as a predictor of depression onset in this population, offering a more practical and efficient approach to depression screening in this specific group.

## Author Contributions

X.C., J.X., L.W., X.L., and G.L. contributed to the conception and design of the study. X.C. contributed to manuscript drafting. X.C., J.X., L.W., H.D., and X.L. contributed to the statistical analysis. H.D. and L.W. contributed to data acquisition. The entire process was supervised by X.L. and G.L. All authors contributed to the critical revisions of the manuscript.

## Conflicts of Interest

The authors declare no conflicts of interest.

## Supporting information


Tables S1.–S3.

## Data Availability

The datasets of this study are available from the HRS and ELSA repositories. The HRS and ELSA data analyzed in this study can be accessed through the following links: https://hrs.isr.umich.edu/and https://www.elsa‐project.ac.uk/.
