# Supplementary material for: Association Between Weight‐Adjusted Waist Index and Depressive Symptoms Among Middle‐Aged and Older Adults: Evidence From Two Prospective Longitudinal Cohort Studies
Source: CNS Neurosci Ther. 2025 Jul 1;31(7):e70496. doi: 10.1111/cns.70496 (PMC12209594; doi:10.1111/cns.70496)
Supplement: Supplementary file 1 — Tables S1.–S3. [file CNS-31-e70496-s001.docx]

**Supplemental Materials**

**Table S1.** Distribution of missing variables in data

| Variables | | **HRS** | |  | **ELSA** | |
| --- | --- | --- | --- | --- | --- | --- |
|  |  | Number of Missing | Missing proportion |  | Number of Missing | Missing proportion |
| Age | | 852 | 25.360% |  | 0 | 0.000% |
| Sex | | 0 | 0.000% |  | 0 | 0.000% |
| Education level | | 0 | 0.000% |  | 0 | 0.000% |
| Marital status | | 0 | 0.000% |  | 93 | 0.028% |
| Smoking status | | 19 | 0.560% |  | 1 | 0.001% |
| Drinking status | | 0 | 0.000% |  | 232 | 7.020% |
| SBP | | 112 | 3.330% |  | 66 | 1.990% |
| DBP | | 128 | 3.810% |  | 47 | 1.423% |
| Height | | 22 | 0.650% |  | 33 | 0.999% |
| BMI | | 22 | 0.650% |  | 33 | 0.999% |
| Hypertension | 0 | | 0.000% |  | 0 | 0.000% |
| Diabetes | 0 | | 0.000% |  | 0 | 0.000% |

Abbreviation: BMI, body mass index; SBP, systolic blood pressure; DBP: diastolic blood pressure.

**TABLE S2A** Baseline characteristics in HRS after excluding individuals with any missing values.

| **Variables** | **HRS** | | | | | |
| --- | --- | --- | --- | --- | --- | --- |
|  |  | **Q1 (n=840)** | **Q2 (n=839)** | **Q3 (n=840)** | **Q4 (n=840)** | ***P*** |
| Age, (years) |  | 66.0 (60.0, 70.0) | 67.0 (63.0, 72.0) | 68.0 (63.0, 74.0) | 70.0 (65.0, 77.0) | < 0.001 |
| Sex, n (%) |  |  |  |  |  | < 0.001 |
| Male |  | 576 (68.6) | 413 (49.2) | 416 (49.5) | 592 (70.5) |  |
| Female |  | 264 (31.4) | 426 (50.8) | 424 (50.5) | 248 (29.5) |  |
| Education level, n (%) |  |  |  |  |  | < 0.001 |
| Junior high school and below |  | 45 (5.4) | 100 (11.9) | 133 (15.8) | 184 (21.9) |  |
| High school or above |  | 795 (94.6) | 739 (88.1) | 707 (84.2) | 656 (78.1) |  |
| Marital status, n (%) |  |  |  |  |  | < 0.001 |
| Other marital status |  | 170 (20.2) | 171 (20.4) | 186 (22.1) | 303 (36.1) |  |
| Married or partnered |  | 670 (79.8) | 668 (79.6) | 654 (77.9) | 537 (63.9) |  |
| Smoking status, n (%) |  |  |  |  |  | < 0.001 |
| No |  | 416 (49.8) | 392 (46.9) | 340 (40.6) | 362 (43.5) |  |
| Yes |  | 419 (50.2) | 443 (53.1) | 497 (59.4) | 471 (56.5) |  |
| Drinking status, n (%) |  |  |  |  |  | < 0.001 |
| No |  | 299 (35.6) | 333 (39.7) | 369 (43.9) | 429 (51.1) |  |
| Yes |  | 541 (64.4) | 506 (60.3) | 471 (56.1) | 411 (48.9) |  |
| SBP, (mmHg) |  | 127.3 (115.3, 141.7) | 126.3 (115.0, 140.2) | 127.7 (115.7, 140.0) | 133.0 (120.4, 146.9) | < 0.001 |
| DBP, (mmHg) |  | 79.0 (71.9, 86.7) | 79.3 (72.3, 87.2) | 79.3 (73.0, 87.0) | 79.0 (71.3, 86.3) | 0.681 |
| Height, (m) |  | 1.7 (1.6, 1.7) | 1.7 (1.6, 1.8) | 1.7 (1.6, 1.7) | 1.6 (1.5, 1.7) | < 0.001 |
| Weight, (Kg) |  | 73.6 (63.5, 85.3) | 80.7 (69.9, 92.9) | 82.8 (72.1, 95.5) | 81.0 (70.1, 94.6) | < 0.001 |
| BMI, (kg/m2) |  | 26.2 (23.6, 29.2) | 28.0 (25.1, 31.3) | 29.4 (26.5, 33.2) | 30.8 (27.6, 35.4) | < 0.001 |
| Waist, (cm) |  | 86.4 (78.7, 94.0) | 96.5 (90.2, 104.1) | 102.9 (96.5, 111.1) | 109.9 (101.6, 119.4) | < 0.001 |
| Hypertension, n (%) |  |  |  |  |  | < 0.001 |
| No |  | 550 (65.5) | 459 (54.7) | 410 (48.8) | 298 (35.5) |  |
| Yes |  | 290 (34.5) | 380 (45.3) | 430 (51.2) | 542 (64.5) |  |
| Diabetes, n (%) |  |  |  |  |  | < 0.001 |
| No |  | 795 (94.6) | 730 (87) | 698 (83.1) | 638 (76) |  |
| Yes |  | 45 (5.4) | 109 (13) | 142 (16.9) | 202 (24) |  |
| WWI |  | 10.1 (9.8, 10.3) | 10.8 (10.7, 10.9) | 11.3 (11.2, 11.4) | 12.1 (11.8, 12.5) | < 0.001 |

Abbreviations: BMI, body mass index; WWI, weight-adjusted waist index; SBP, systolic blood pressure;

DBP, diastolic blood pressure

**TABLE S2B** Baseline characteristics in ELSA after excluding individuals with any missing values.

| **Variables** | **ELSA** | | | | |
| --- | --- | --- | --- | --- | --- |
|  | **Q1 (n= 826)** | **Q2 (n= 825)** | **Q3 (n= 826)** | **Q4 (n= 826)** | ***P*** |
| Age, (years) | 61.0 (56.0, 67.0) | 61.0 (57.0, 69.0) | 63.0 (57.0, 70.0) | 67.0 (60.0, 74.0) | < 0.001 |
| Sex, n (%) |  |  |  |  | < 0.001 |
| Male | 628 (76) | 477 (57.8) | 376 (45.5) | 366 (44.3) |  |
| Female | 198 (24) | 348 (42.2) | 450 (54.5) | 460 (55.7) |  |
| Education level, n (%) |  |  |  |  | < 0.001 |
| Junior high school and below | 191 (26) | 246 (32.7) | 299 (38.8) | 387 (51.6) |  |
| High school or above | 543 (74) | 507 (67.3) | 471 (61.2) | 363 (48.4) |  |
| Marital status, n (%) |  |  |  |  | 0.219 |
| Other marital status | 207 (26) | 207 (25.9) | 196 (24.5) | 235 (28.9) |  |
| Married or partnered | 590 (74) | 593 (74.1) | 605 (75.5) | 577 (71.1) |  |
| Smoking status, n (%) |  |  |  |  | < 0.001 |
| No | 396 (47.9) | 347 (42.1) | 299 (36.2) | 271 (32.8) |  |
| Yes | 430 (52.1) | 478 (57.9) | 527 (63.8) | 554 (67.2) |  |
| Drinking status, n (%) |  |  |  |  | 0.019 |
| No | 55 (7) | 52 (6.7) | 49 (6.4) | 76 (10.2) |  |
| Yes | 729 (93) | 722 (93.3) | 718 (93.6) | 670 (89.8) |  |
| SBP, (mmHg) | 74.0 (68.0, 81.0) | 75.7 (68.7, 83.0) | 77.3 (70.2, 84.7) | 76.7 (69.0, 84.0) | < 0.001 |
| DBP, (mmHg) | 110.0 (102.0, 119.3) | 112.7 (103.3, 122.6) | 115.7 (106.7, 126.3) | 116.0 (107.3, 126.7) | < 0.001 |
| Height, (m) | 1.6 (1.6, 1.7) | 1.7 (1.6, 1.7) | 1.7 (1.6, 1.7) | 1.6 (1.6, 1.7) | < 0.001 |
| Weight, (Kg) | 68.0 (60.7, 77.4) | 75.2 (65.7, 84.5) | 79.0 (70.4, 88.9) | 81.0 (71.1, 92.5) | < 0.001 |
| BMI, (kg/m2) | 24.9 (22.7, 27.1) | 26.7 (24.6, 29.5) | 28.1 (25.8, 31.2) | 29.8 (26.7, 32.9) | < 0.001 |
| Waist, (cm) | 82.3 (76.6, 88.3) | 92.2 (86.2, 98.2) | 98.9 (93.0, 105.2) | 105.5 (98.8, 113.4) | < 0.001 |
| Hypertension, n (%) |  |  |  |  | < 0.001 |
| No | 631 (76.4) | 548 (66.4) | 498 (60.3) | 434 (52.5) |  |
| Yes | 195 (23.6) | 277 (33.6) | 328 (39.7) | 392 (47.5) |  |
| Diabetes, n (%) |  |  |  |  | < 0.001 |
| No | 804 (97.3) | 791 (95.9) | 774 (93.7) | 730 (88.4) |  |
| Yes | 22 (2.7) | 34 (4.1) | 52 (6.3) | 96 (11.6) |  |
| WWI | 9.9 ± 0.4 | 10.7 ± 0.1 | 11.1 ± 0.1 | 11.8 ± 0.4 | < 0.001 |

Abbreviations: BMI, body mass index; WWI, weight-adjusted waist index; SBP, systolic blood pressure;

DBP, diastolic blood pressure

| **Table S3.** Association between the WWI and depression after excluding individuals with any missing values. | | | | | | |
| --- | --- | --- | --- | --- | --- | --- |
| **(HRS) Variables** | **Model 1** | | **Model 2** | | **Model 3** | |
|  | **HR (95% CI)** | ***P*** | **HR (95% CI)** | ***P*** | **HR (95% CI)** | ***P*** |
| WWI | 1.35 (1.29~1.42) | <0.001 | 1.24 (1.17~1.32) | <0.001 | 1.15 (1.08~1.23) | <0.001 |
| 1st Quartile (<10.520) | 1(Reference) |  | 1(Reference) |  | 1(Reference) |  |
| 2nd Quartile (10.520–11.041) | 1.19 (1.03~1.37) | 0.015 | 1.25 (1.06~1.48) | 0.01 | 1.16 (0.98~1.38) | 0.088 |
| 3rd Quartile (11.041–11.606) | 1.51 (1.32~1.72) | <0.001 | 1.46 (1.24~1.72) | <0.001 | 1.30 (1.10~1.55) | 0.003 |
| 4th Quartile (≥11.606) | 2.06 (1.85~2.35) | <0.001 | 1.79 (1.53~2.09) | <0.001 | 1.48 (1.25~1.76) | <0.001 |
| p for trend | 1.28 (1.23~1.33) | <0.001 | 1.21 (1.15~1.27) | <0.001 | 1.14 (1.08~1.20) | <0.001 |
|  | | | | | | |
| **(ELSA) Variables** | **Model 1** | | **Model 2** | | **Model 3** | |
|  | **HR (95% CI)** | ***P*** | **HR (95% CI)** | ***P*** | **HR (95% CI)** | ***P*** |
| WWI | 1.33 (1.24~1.42) | <0.001 | 1.25 (1.17~1.34) | <0.001 | 1.20 (1.11~1.30) | <0.001 |
| 1st Quartile (<10.394) | 1(Reference) |  | 1(Reference) |  | 1(Reference) |  |
| 2nd Quartile (10.394–10.89) | 1.05 (0.92~1.20) | 0.489 | 1.08 (0.94~1.24) | 0.295 | 1.10 (0.95~1.27) | 0.222 |
| 3rd Quartile (10.89–11.374) | 1.18 (1.03~1.35) | 0.017 | 1.22 (1.07~1.40) | 0.004 | 1.20 (1.03~1.39) | 0.021 |
| 4th Quartile (≥11.374) | 1.62 (1.43~1.85) | <0.001 | 1.50(1.31~1.71) | <0.001 | 1.38 (1.18~1.61) | <0.001 |
| p for trend | 1.18 (1.13~1.23) | <0.001 | 1.15 (1.10~1.20) | <0.001 | 1.11 (1.06~1.17) | <0.001 |

Abbreviation: WWI, weight-adjusted waist index; SBP, systolic blood pressure; DBP, diastolic blood pressure.

*Note:* Model 1: unadjusted.

Model 2: adjusted for age and sex.

Model 3: adjusted for Model 1 + education level, marital status, smoking status, drinking status, SBP, DBP, height, diabetes.
